# Editorial: Neuroinflammation and affective/cognitive impairment: The role of white matter and glial cells

**DOI:** 10.3389/fnagi.2022.1115180

**Published:** 2023-01-05

**Authors:** Bingying Du, Yanbo Zhang, Xiaoying Bi

**Affiliations:** ^1^Department of Neurology, Shanghai Changhai Hospital, The Second Military Medical University, Shanghai, China; ^2^Department of Psychiatry, Faculty of Medicine and Dentistry, University of Alberta, Edmonton, AB, Canada

**Keywords:** cognitive impairment, inflammation, white matter, glial, cerebral small-vessel disease (CSVD)

In recent years, neuroinflammatory changes have been increasingly recognized as potential mediators of a wide range of neurological diseases, including cerebrovascular diseases (Shi et al., 2019; Jurcau and Simion, 2021), depression (Jia et al., 2021), multiple sclerosis (Voet et al., 2019), Alzheimer's disease (Calsolaro and Edison, 2016; Mangalmurti and Lukens, 2022), and Parkinson's disease (PD) (Badanjak et al., 2021). Neuroinflammation occurs before visible structural damage to the brain appears. Given the high complexity of the aging process and various mechanisms contributing to cognition/emotion-related diseases, it is arduous to pinpoint any single factor. This Research Topic in Frontiers in Aging Neuroscience has produced a highly informative collection of six original research articles and one review, comprehensively covering neuroinflammation and novel interventions in rescuing affective or cognitive impairments.

The term “Vascular Cognitive Impairment (VCI)” was introduced in recent years to encapsulate the whole spectrum of disease, ranging from subjective cognitive impairment and mild cognitive impairment (MCI) to dementia associated with underlying cerebrovascular disease burden (O'Brien et al., 2003; van der Flier et al., 2018). Neuroimaging of VCI includes large infarcts, small and lacunar infarcts, white matter lesions (WMLs), microbleeds, enlarged perivascular spaces, global atrophy, the structural integrity of the white matter, and cortical microinfarcts (van der Flier et al., 2018). Cerebral small-vessel disease (CSVD) is the leading cause of VCI, and amyloid β-protein (Aβ) deposition is one of the most significant pathologies of CSVD-induced VCI (Held et al., 2017). A study by Liu et al. confirms the essential roles of aquaporin 4 (AQP4) polarity and neuroinflammation activation in CSVD, which are critical regulators for Aβ clearance and production, respectively. They found that treatment with mesenchymal stem cells (MSCs) markedly reduced Aβ deposition in CSVD animal models by both promoting AQP4 polarity and alleviating neuroinflammation through the STAT6 pathway while failing to reduce WMLs. The study by Song et al. extends the term VCI to include network disconnection disorders. They were dedicated to investigating alterations of glymphatic function in VCI and its potential impact on network connectivity by DTI-ALPS, a novel imaging approach. Their results indicate that glymphatic dysfunction might affect cognitive function in VCI by disrupting network connectivity and may be a potential common pathological mechanism of VCI. As for the early stages of VCI, Zhang et al. emphasize the role of green tea consumption in early MCI intervention. They found that regular green tea consumption is associated with better cognitive function among Chinese middle-aged and older adults, especially in memory and executive function. Meanwhile, higher levels of tea consumption have a more substantial protective effect, which might be achieved by reducing AD-related pathology and improving anti-oxidative stress capacity. These three studies in our Research Topic provide a comprehensive insight into the pathogenesis of VCI from molecular and imaging perspectives. Furthermore, they offer evidence regarding the benefits of green tea on cognitive function.

Definitive evidence has shown that MCI is commonly comorbid with depression (Ismail et al., 2017). As a high-incidence-rate mental disease, depression is reported to influence around 5% of adults globally (Herrman et al., 2022). It is a heterogeneous disease, and the molecular pathology involved in the development of depression is complex, with neuroinflammation and WMLs rarely being seen in depression patients (Wang et al., 2014; Won et al., 2021). However, the neural mechanisms underlying the reduced emotion regulation in individuals with MCI combined with depressive symptoms are not precise. In a study utilizing event-related electroencephalography (EEG), Liu et al. explore the relationship between depression with cognition and emotion regulation and how depressive symptoms affect emotion preprocessing and its potential mechanism. The results show that MCI-depressed individuals prefer negative stimuli, and the severity of depressive symptoms highly correlates with the ability to regulate emotion. Most importantly, MCI in depressed individuals exhibited a higher tendency toward processing negative stimuli because of increased low-frequency oscillations and decreased high-frequency activity. Therefore, it is speculated that these might be important factors contributing to the impairment of emotion regulation in MCI-depressed individuals. In addition to the neural circuitry of depression, signaling pathways and transcription factors in the process of depression have also not been clarified. Considering the characteristics of multi-components and multi-targets of traditional Chinese medicine (TCM), He et al. comprehensively discuss the advances in the field in support of active constituents on the important targets (CREB, NF-κB, and Nrf2) and the relevant signaling pathways (BDNF-TrkB, MAPK, GSK-3β, and TLR-4) for the prevention and treatment of depression. Their contributions provide a basis for researching and developing novel antidepressants characterized by rapid action.

Stroke is the second leading cause of death worldwide and the leading cause of VCI (Wu et al., 2019). The last decades have seen the rapid expansion of research on post-stroke cognitive impairment and depression. However, much less attention has been paid to post-stroke fatigue (PSF), which occurs about as frequently as cognitive impairment and depression after stroke (Cumming et al., 2016). In the study conducted by Wang et al., in a group of 361 patients with acute stroke, lesions in the right thalamus were identified by voxel-based lesion-symptom mapping, which increase the risk of fatigue symptoms 6 months post-stroke. This result deserves the attention of clinicians; that is, when an ischemic lesion located in the right thalamus is detected, particular attention should be paid to the occurrence of PSF.

Parkinson's disease (PD) is the second most common neurodegenerative disorder, affecting >1% of the population ≥65 years of age (Aarsland et al., 2021). In addition to the defining motor symptoms, multiple non-motor symptoms occur in PD; cognitive impairment being the most common (Aarsland et al., 2021). Not only does it concern clinical symptoms (such as cognitive impairment, emotional disorders, and gait dysfunction), PD and CSVD have similarities with *in vivo* imaging, for example, WMLs (de Schipper et al., 2019; Wei et al., 2021). Therefore, vascular pathology is naturally thought to be a major culprit of cognitive decline in PD patients. Similarly, Hou et al. conducted a retrospective study to evaluate the possibility of utilizing the CSVD burden [cored based on lacunes, enlarged perivascular spaces (EPVS), high-grade white matter hyperintensities (WMH), and cerebral microbleeds (CMBs)] as the independent predictor of cognitive decline in PD in the clinical setting. They found that educational level, PD questionnaire 39 (PDQ39), and CSVD burden were significantly associated with Montreal Cognitive Assessment (MoCA) scores in individuals with PD, and cognitive scores of languages, delayed recall, and orientation were significantly reduced in PD patients with CSVD burden ≥1 compared to those with CSVD burden = 0. Their contributions suggest that promoting neurovascular health may help preserve cognitive functions in PD.

By its diverse contributions, this Research Topic collates a group of studies mainly on molecular and structural or functional changes revealed by *in vivo* imaging or EEG on affective or cognitive impairment. We hope this Research Topic has created a forum for in-depth discussions that help to better understand the inherent mechanisms in brain aging and the progression of neurodegeneration.

## Author contributions

XB conceived and designed the Research Topic. BD summarized the relevant articles and drafted the manuscript that was reviewed and edited by YZ. All authors have participated and made substantial contributions to this paper. All authors contributed to the article and approved the submitted version.
